# Heavy Metal Adsorption onto *Kappaphycus* sp. from Aqueous Solutions: The Use of Error Functions for Validation of Isotherm and Kinetics Models

**DOI:** 10.1155/2015/126298

**Published:** 2015-07-30

**Authors:** Md. Sayedur Rahman, Kathiresan V. Sathasivam

**Affiliations:** Faculty of Applied Sciences, AIMST University, 08100 Bedong, Kedah, Malaysia

## Abstract

Biosorption process is a promising technology for the removal of heavy metals from industrial wastes and effluents using low-cost and effective biosorbents. In the present study, adsorption of Pb^2+^, Cu^2+^, Fe^2+^, and Zn^2+^ onto dried biomass of red seaweed *Kappaphycus* sp. was investigated as a function of pH, contact time, initial metal ion concentration, and temperature. The experimental data were evaluated by four isotherm models (Langmuir, Freundlich, Temkin, and Dubinin-Radushkevich) and four kinetic models (pseudo-first-order, pseudo-second-order, Elovich, and intraparticle diffusion models). The adsorption process was feasible, spontaneous, and endothermic in nature. Functional groups in the biomass involved in metal adsorption process were revealed as carboxylic and sulfonic acids and sulfonate by Fourier transform infrared analysis. A total of nine error functions were applied to validate the models. We strongly suggest the analysis of error functions for validating adsorption isotherm and kinetic models using linear methods. The present work shows that the red seaweed *Kappaphycus* sp. can be used as a potentially low-cost biosorbent for the removal of heavy metal ions from aqueous solutions. Further study is warranted to evaluate its feasibility for the removal of heavy metals from the real environment.

## 1. Introduction

Heavy metal pollution due to rapid urbanization and industrialization is one of the most significant environmental problems. Heavy metals are released into the aquatic environment from several domestic (automobile exhaust, smelting processes, burning of fossil fuels, incineration of wastes, landfill leaches, use of sewage sludge, municipal wastewater, and urban runoff) and industrial processes (electroplating, refining ore, mining, electronic and metal-finishing industries, fertilizer industry, tanneries, painting, paper industries, and pesticides) [1]. Heavy metals have become a global issue of environment and public health concern due to their toxicities, bioaccumulation in human body and food chain, carcinogenicities, and mutagenesis in various living organisms [2–4].

Numerous methods such as chemical precipitation, ion exchange, coagulation-flocculation, flotation, membrane filtration, electrochemical treatment, magnetic separation and purification, biosorption, and nanotechnology are being used to treat or remove heavy metals from water and wastewater [1, 5, 6]. Among them, biosorption has been regarded as a promising cost-effective, sustainable, and ecofriendly technology for the removal of different types of organic and inorganic pollutants from water and wastewater [7]. Moreover, this process offers a number of advantages in comparison to the conventional methods [8].

A wide range of commercial and potentially low-cost adsorbents including living or dead microorganisms, seaweeds, plant materials, industrial and agricultural wastes, natural residues, and inorganic precursors including red mud, clays, blast furnace slags, zeolites, chitosan, and peat has been reported in literature [8–12]. Seaweeds are widely distributed in marine, freshwater, and terrestrial ecosystems, which can serve as good biosorbents due to their abundance, cost-effectiveness, reusability, and high metal sorption capacities [7, 10, 11]. Despite that fact that the red algae constitute carrageenan that provides different binding sites (e.g., hydroxyl, carboxyl, amino, and sulfhydryl) responsible for the adsorption for heavy metals [9], they are the least focused group [13]. Therefore, further research studies are warranted on the selectivity of algal species [9].

The red seaweed* Kappaphycus* sp. is one of the most important commercial sources of kappa-carrageenan, which also has different medicinal and industrial applications [14]. Malaysia produced 331,490 tonnes of* Kappaphycus* sp., being 17.039% of the total world production in 2012 [15]. Recent studies suggest that both the living biomass and the waste biomass of* Kappaphycus alvarezii* are a good biosorbent for the removal of nutrients [13] and heavy metals from the aqueous environment [16, 17]. However, there is no available literature report on the biosorption of heavy metals using the dry biomass of* Kappaphycus* sp. Hence, the present study was investigated to study the performance of the dried biomass of* Kappaphycus* sp. for the removal of Zn^2+^, Cu^2+^, Pb^2+^, and Fe^2+^ from aqueous solutions in batch system at laboratory scale under different parameters like pH of solution, contact time, temperature, and initial metal ion concentrations. The Fourier transform infrared (FTIR) spectral analysis was made to identify the main functional groups involved in the biosorption process of those metal ions.

In general, mechanistic or empirical equations are used to express heavy metal adsorption capacities of different types of biosorbents using batch or column method [18]. Available literature reports confirm that nearly two dozens of empirical models involving 2, 3, 4, or even 5 parameters have been used to fit batch equilibrium isotherm curves to biosorbents [18, 19]. Besides, kinetic models have been described by several authors elsewhere [20–22]. The equilibrium and kinetic models are often validated on the basis of coefficient of regression (*R* ^2^ ≥ 0.99) of the experimental data. In the present study, some error functions have been used for validating the experimental data along with an insight into the usual measures of model inferences.

## 2. Materials and Methods

### 2.1. Collection, Identification, and Preparation of the Biosorbent

The red seaweed* Kappaphycus* sp. was collected from the Semporna coast of Sabah, Malaysia, in April 2013. The alga was washed for several times with running water and subsequently with deionized water to remove epiphytes and salts. The washed biomass was then dried in an oven at 60°C for 48 h until a constant weight was attained. The dried biomass was then crushed with an analytical mill, sieved (250 *μ*m size), and stored in polypropylene bottles until use.

The living biomass of the species was preliminarily identified following systemic morphological features [23]. It was then subjected to 28S DNA based molecular identification [24]. The species was identified as* Kappaphycus* sp. and the gene sequence of the nucleotide was submitted in the NCBI GenBank (accession number KM229320).

### 2.2. Chemicals and Reagents

All the chemicals and reagents used in this study were of analytic reagent grade. The working solutions of different concentrations (10–200 mg L^−1^) of the heavy metals (Zn, Cu, Pb, and Fe) were prepared by diluting the stand solutions (1000 ± 2 mg L^−1^) of the metals (Merck, Germany) in double distilled deionized water. Different initial pH of the solutions was obtained by adding 0.1 N HCl (Sigma-Aldrich, USA) or 0.1 N NaOH (Merck, Germany).

### 2.3. Batch Biosorption Experiments

All the experiments were conducted in a batch system using 150 mL Erlenmeyer flasks in a thermostatic shaker (25°C, 180 rpm), unless otherwise stated. Each flask was filled with 50 mL of solution and biosorbent as appropriate. The influence of several operational parameters on the biosorption characteristics of the metals such as pH of the aqueous solution (2–7), contact time (0−120 min), initial metal ion concentration (25−200 mg L^−1^), and temperature (25−50°C) were assessed using a constant biomass dosage (4 g L^−1^). Competitive adsorption of the four metal ions under mixed condition was also evaluated.

The adsorption studies were conducted with 50 mL of the metal solutions at an initial concentration of 10 mg L^−1^. For the kinetic studies sample solutions were withdrawn at regular intervals and the residual concentration of the heavy metals in the aqueous phase was analyzed after filtration as stated above.

The amount of the metal ions remaining in the solutions was measured by using Atomic Absorption Spectrometer (AAnalyst700, Perkin-Elmer, USA) after separation of the biosorbent by filtration through Whatman Filter number 1.

The amount of metal adsorbed per gram of the biosorbent at equilibrium, *q*_*e*_ (mg g^−1^), was calculated from the difference of the metal concentration in the aqueous phase before and after biosorption as follows:

(1) $$\begin{array}{c} q_{e}=\frac{\left(C_{0}-C_{e}\right)\times V}{m}, \end{array}$$

where *C*_0_ and *C*_*e*_ are the initial and equilibrium concentration of metal ions in the solution (mg/L), respectively, *V* is the volume of metal solution (L), and *m* is the mass of the dry biosorbent (g).

The percentage of metal removal (*R*, %) from the solution was calculated as follows:

(2) $$\begin{array}{c} R\left(\;\%\;\right)=\frac{\left(C_{0}-C_{e}\right)\times 100}{C_{0}}. \end{array}$$

Each experiment was done in triplicate and the data were expressed as the mean of the triplicate results. Statistical analyses were performed using Microsoft Office Excel 2007 (Microsoft Corp., USA).

### 2.4. Application of Adsorption Models

In the present experiment, four two-parameter isotherm models: Langmuir, Freundlich, Temkin, and Dubinin-Radushkevich (D–R); four two-parameter kinetic models: pseudo-first-order (PFO), pseudo-second-order (PSO), Elovich, and intraparticle diffusion (IpD); and kinetic model were applied to describe the sorption behaviour of the adsorbent. The equations of these models are given in Table 1.

### 2.5. Error Function Analysis

In order to evaluate the suitability of the equation to the experimental data error function is the best optimization procedure. Apart from the regression coefficient (*R* ^2^), nine error functions such as sum of square error (*SSE*), average relative error (*ARE*), hybrid functional error (*HYBRID*), sum of absolute error (*EABS*), Marquardt's percent standard deviation (*MPSD*), normalized standard deviation (Δ*q*(%)), coefficient of determination (*r* ^2^), nonlinear chi-square test (*χ* ^2^), and residual root mean square error (*RMSE*) were calculated to evaluate the best fit of the modeled equation to the experimental data. The equations of the error functions are given in Table 2.

### 2.6. FTIR Analysis

FTIR spectral analysis was carried out to determine the possible functional groups present in the dried biomass of* Kappaphycus* sp. Infrared spectra of the raw and metal-loaded biomass were obtained using a Fourier transform infrared (FTIR) spectrometer (Spectrum GX, Perkin-Elmer, USA).

## 3. Results and Discussion

### 3.1. Effect of Solution pH

**Figure 1:**
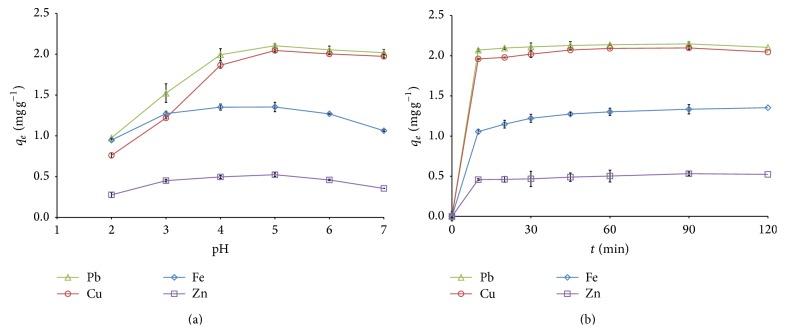
(a) Effect of solution pH on the metal ions biosorption onto* Kappaphycus* sp. dried biomass. (b) Effect of contact time on the metal ions biosorption onto* Kappaphycus* sp. dried biomass.

In the adsorption process of metal ions from aqueous solutions, pH of the solution plays an important role. It is apparent from the results represented graphically in Figure 1(a) that, with the increase in pH, the biosorption increased gradually. The maximum biosorption (54.13%, 81.84%, 84.17%, and 20.94% for Zn^2+^, Cu^2+^, Pb^2+^, and Fe^2+^, resp.) was observed at pH 5. At lower pH (2–4), biosorption of metal ions was inhibited greatly. This can be explained on the basis that cell wall of the* Kappaphycus* sp. contains various functional groups (as described in Section 3.9). The positively charged functional groups increase competition between protons and metal cations for binding active sites of biomass, resulting in decreasing the metal cations adsorption on the biomass surfaces [48]. At higher pH values (6–8), the biosorption efficiency of metal ions was significantly decreased (Figure 1(a)), which may be attributed to the formation of anionic hydroxide complexes that decrease the dissolved metal concentration in solution and their competition with the active sites [49]. Therefore, all the rest biosorption experiments were carried out at pH 5.

### 3.2. Effect of Contact Time

As shown in Figure 1(b), biosorption of metal ions on the adsorbent increased with an increase in contact time and the equilibrium biosorption was attained within 90–120 min reflecting rapid initial biosorption. Maximum uptake of Cu^2+^, Pb^2+^, Fe^2+^, and Zn^2+^ was reached up to 83.88, 85.89, 21.27, and 54.13%, respectively, within 90 min. A decrease in the biosorption was noticed during the subsequent time of incubation indicating the maximum adsorption level as a saturation point of biosorption. The rapid kinetic mechanism can be attributed to the formation of exterior surface complexes neglecting intraparticle diffusion, which is very advantageous in biotechnological processes for wastewater treatment [48]. In general, heavy metal biosorption efficiency of seaweeds attained a maximum level within 30 and 90 min [50]. Hence, a contact time of 120 min was selected for further experiments ensuring attainment of equilibrium conditions.

### 3.3. Effect of Temperature

The solution temperature plays a vital role on the metal ions biosorption, which was found to increase with the increase of solution temperature. The rate of Zn^2+^, Cu^2+^, Pb^2+^, and Fe^2+^ biosorption by the dried biomass of* Kappaphycus* sp. was rapid reaching a maximum of 61.74, 89.12, 86.68, and 40.06%, respectively, at 50°C. This phenomenon indicates that the biosorption process of the metal ions onto the biomass is endothermic. It can be attributed that, at the higher temperatures, the activation of the biosorbent surfaces is enlarged facilitating more active sites for biosorption of the metal ions. Moreover, an easy mobility and enhanced accessibility of metal ions from the bulk solution to the biomass active sites could also be the possible reason for the maximum biosorption of metal ions at higher temperatures [51].

### 3.4. Effect of Initial Metal Ion Concentration

Biosorption capacity of the biomass was found to increase with increasing initial concentration of the metal ions. This phenomenon can be attributed to an increase in electrostatic interactions involving sites of progressively lower affinity for the metal ions up to the point of saturation [51, 52]. It was further observed that the percentage removal of the metal ions decreased markedly from 77.52% to 30.58% for Zn^2+^, 87.52% to 36.09% for Cu^2+^, 87.12% to 40.04% for Pb^2+^, and 80.72% to 31.52% for Fe^2+^ with an increase in the initial concentration of the metal ions from 25 to 200 mg L^−1^. This might be due to the rapid saturation of all metal binding active sites of the biosorbent at a certain concentration of the metal ions [52, 53] and an equilibrium state between adsorbate and biosorbent was attained.

### 3.5. Biosorption Isotherm Studies

The equilibrium adsorption isotherms are essential data source to design, understand, and optimize the biosorption process. The data express the intrinsic properties of the biosorbent and interaction between adsorbate and adsorbent. The data can be used to compare the biosorptive capacities of the biosorbent for different pollutants.

#### 3.5.1. Langmuir Isotherm Model

**Figure 2:**
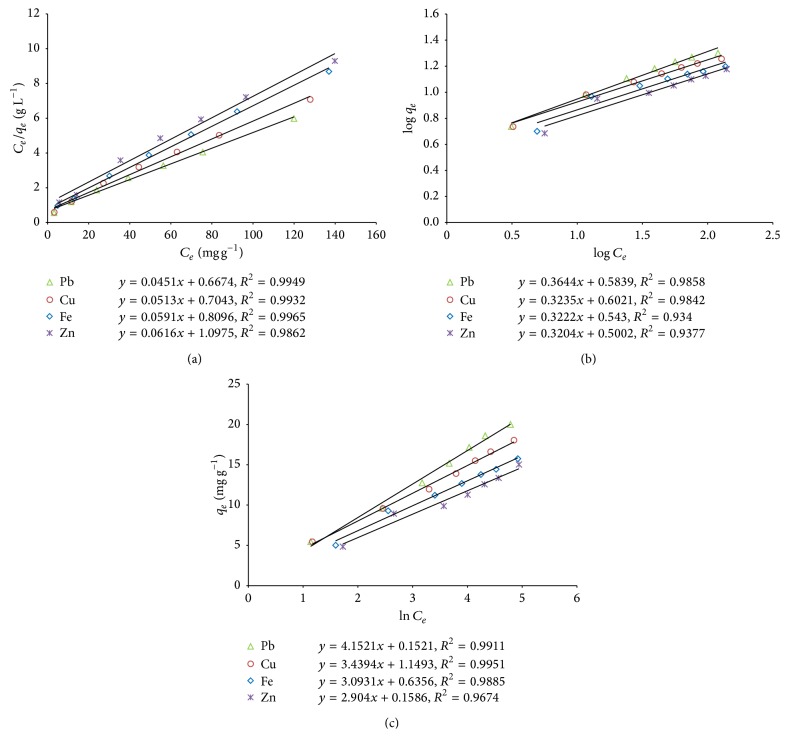
(a) Langmuir model for the metal ions biosorption onto* Kappaphycus* sp. dried biomass. (b) Freundlich model for the metal ions biosorption onto* Kappaphycus* sp. dried biomass. (c) Temkin model for the metal ions biosorption onto* Kappaphycus* sp. dried biomass.

The model isotherm parameters together with regression coefficient are represented in Table 3. As shown in Figure 2(a), approximately linear relationship (*R* ^2^ ≥ 0.99) exists in the adsorption isotherms for* Kappaphycus* sp. The maximum Langmuir monolayer adsorption capacity of the sorbent, *q*_*m*_ (mg g^−1^), for the experimental metal ions followed an increasing order: Pb^2+^ (22.27) > Cu^2+^ (19.46) > Fe^2+^ (17.09) > Zn^2+^ (16.78), suggesting that Pb^2+^ has a preferential uptake compared to the other metals, which can be attributed to its low tendency in forming strong complex [46]. Another reason might be attributed to carboxylate polysaccharides in seaweeds that show preferential binding of cations with large ionic radii [48]. However, the preferential sorption order of the metal ions in the present study can be explained by Pauling's electronegativity [54]: Pb^2+^ (2.33) > Cu^2+^ (1.190) > Fe^2+^ (1.83) > Zn^2+^ (1.65). This implies the fact that the higher the ion's electronegativity the higher the attraction for its electrons, and the attraction becomes stronger to the negative charge of the biomass ligands [55]. Furthermore, the separation factor, *R*_*L*_, for the metal ions (Table 3) falls within the range of 0 < *R*_*L*_ < 1 suggesting that adsorption of the experimental ions is favourable at all the concentrations investigated [26]. Hence, the* Kappaphycus* sp. is a suitable biosorbent for the sorption of the experimental metal ions from aqueous solutions.

A comparative study on maximum heavy metal adsorption capacity of different low-cost adsorbents has been given in Table 4. The study shows that the dried biomass of* Kappaphycus* sp. is more promising than some other low-cost adsorbents for the removal of the metal ions.

#### 3.5.2. Freundlich Isotherm Model

The linearized Freundlich isotherm model is shown in Figure 2(b); Freundlich constants *K*_*F*_ and *n* are represented in Table 3. The results suggest that the biosorption of Pb^2+^ can be moderately described by the Freundlich model (*R* ^2^ ≈ 0.99). The magnitude of Freundlich isotherm constant, *K*_*F*_, suggests that the sorption capacity of the experimental metal ions was in the order of Cu^2+^ > Pb^2+^ > Fe^2+^ > Zn^2+^. The values of *n* > 1 suggest heterogeneity of the biomass surface, and the metal ions are favourably and intensively biosorbed by the dried biomass of* Kappaphycus* sp. under the experimental conditions.

#### 3.5.3. Temkin Isotherm Model

The Temkin isotherm model indicates the adsorption potentials of the adsorbent for adsorbates. The Temkin isotherm plots (Figure 2(c)) and parameters (Table 3) indicate that the model fits the experimental data well (*R* ^2^ ≥ 0.99) for describing the metal ions (Pb^2+^, Cu^2+^, and Fe^2+^) adsorption. The lower values of the Temkin adsorption potential, *A*_*T*_ (L mg^−1^), in the range of 0.769 to 1.455 indicate a lower sorbent-metal ion potential. Furthermore, the lower values (0.585–0.792) of the Temkin constant *b*_*T*_ (kJ mol^−1^) indicate a weak sorbate-sorbent interaction [45].

#### 3.5.4. D-R Isotherm Model

The D-R isotherm parameters (Table 3) indicate that the D-R model does not fit the experimental data well (*R* ^2^ ≤ 0.89) for describing the metal ions biosorption, suggesting the involvement of metal sorption mechanisms other than van der Waals force [56]. The mean free energy of biosorption, *E* (0.297−0.489 kJ·mol^−1^), for the metal ions suggests that the sorption process is physisorption [57] and corroborative to the earlier reports in literature [46, 48]. The positive values of *E* indicate the endothermic nature of the sorption process [46]. Furthermore, the values of *E* (<16 kJ mol^−1^) suggest that the mechanism of the ion exchange process is film-diffusion controlled [58].

#### 3.5.5. Competitive Adsorption

Competitive adsorption of the metal ions under quaternary system shows the adsorption preference of Pb^2+^ > Cu^2+^ > Fe^2+^ > Zn^2+^ with the rate of metal removal as 90.39, 90.00, 75.00, and 58.38%, respectively. The results suggest that the potentiality of the adsorbent in the quaternary system remains the same as that in the single metal system, which proves its unique adsorption quality.

### 3.6. Biosorption Kinetic Studies

The kinetic data are essential to understand the rate and nature of adsorption onto the adsorbents. The data can be used to compare the kinetics of the biosorbent for different pollutants.

#### 3.6.1. Pseudo-First-Order Kinetic Studies

**Figure 3:**
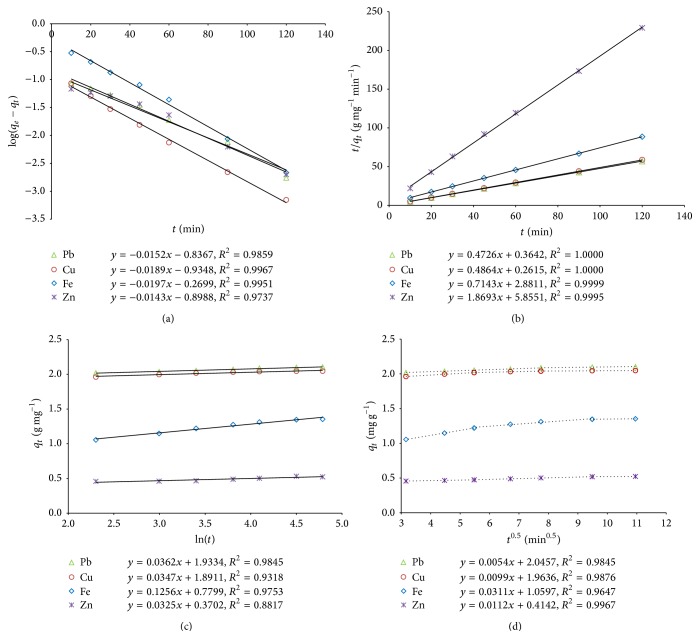
(a) Pseudo-first-order model for the metal ions biosorption onto* Kappaphycus* sp. dried biomass. (b) Pseudo-second-order model for the metal ions biosorption onto* Kappaphycus* sp. dried biomass. (c) Elovich model for the metal ions biosorption onto* Kappaphycus* sp. dried biomass. (d) Intraparticle diffusion model for the metal ions biosorption onto* Kappaphycus* sp. dried biomass.

As shown in Table 5 and Figure 3(a), the regression coefficient (*R* ^2^ ≥ 0.99) of the pseudo-first-order model suggests that the experimental data accurately support the PFO model to describe adsorption kinetics of the metal ions. But the differences between the experimental values, *q*_*e*_, were higher than the modelled values, *q*_*m*_. It refers to the fact that both the metal ions and adsorbent were involved in the adsorption process [52]. Therefore, it is suggested that the pseudo-first-order model is not suitable to explain the kinetic sorption of the experimental metal ions onto the dried biomass of* Kappaphycus* sp. over the range of experimental time and metal ion concentrations. Similar results have been reported for the sorption kinetic of different metal ions onto different adsorbents including seaweeds in the literature [51, 52, 59, 60].

#### 3.6.2. Pseudo-Second-Order Kinetic Model

The values of the regression coefficient of the linearized PSO kinetic model as shown in Figure 3(b) were the highest (*R* ^2^ > 0.99) among the studied kinetic models, and the experimental *q*_*e*_ values matched well with the calculated data (Table 5). Therefore, it can be suggested that the experimental data accurately support the best fit of the PSO model for the adsorption of the metal ions. Hence, chemisorption is the rate-limiting step which involves valence forces through the sharing or exchange of electrons between the metal ions and different functional groups in the sorbent [13, 60].

The pseudo-second-order rate constant, *k*_2_ (g mg^−1^ min^−1^), was found in the range of 0.1874 to 0.9548, which supports that the metal ions uptake onto the sorbent from aqueous solution was more rapid and favourable. As shown in Figure 3(b), adsorption kinetic of the metal ions on* Kappaphycus* sp. occurred in two steps: a fast initial uptake rate, *h* (0.17–3.82 mg g^−1^ min^−1^), in the first 30 min, where more than 85% of the total metal adsorption occurred, followed by a slower uptake rate leading to the equilibrium state (~120 min). Similar observation was reported in literature [51, 52]. This phenomenon supports that the diffusion is the rate-controlling step in the sorption process [60]. The half-adsorption time *t*_1/2_ (min) defined as the time required for the adsorption to take up half amount of the equilibrium metal ions was found within the range of 0.54 to 4.03 indicating high affinity between the adsorbate and adsorbent molecules [61].

#### 3.6.3. Elovich Model

The values of the regression coefficient (*R* ^2^ = 0.79–0.98) of the Elovich kinetic model (Table 5, Figure 3(c)) suggest that kinetic data did not follow the Elovich model. However, the higher values of the Elovich constants, *α* (mg g^−1^ min^−1^) and *β* (g mg^−1^), as shown in Table 5 are suggestive of an increased rate of chemisorption [13].

#### 3.6.4. Intraparticle Diffusion Model

The nonlinear regression data of *q*_*t*_ versus *t* ^0.5^ plots as shown in Figure 3(d) for different heavy metal ions suggests multilinearity (two phases in Pb^2+^ adsorption and three phases in Cu^2+^, Fe^2+^, and Zn^2+^ adsorption). The intraparticle diffusion rate constant (*k*_*d*_) as shown in Table 5 was obtained from the slope of the second linear portions of the plot of *q*_*t*_ versus *t* ^0.5^ for the metal ions. Apparently intraparticle diffusion plays a significant role in the adsorption of Pb^2+^, Cu^2+^, and Zn^2+^ (*R* ^2^ ≥ 0.99) onto the dried biomass of* Kappaphycus* sp. suggesting the fact that there is a significant relationship between *q*_*t*_ and *t* ^0.5^ for the metal ions at the experimental conditions. However, *q*_*t*_ versus *t* ^0.5^ plots did not pass through the origin (*C* > 0) in any of the cases, suggesting that even though the adsorption process involved intraparticle diffusion, it was not the only rate-controlling step [13, 62], and external mass transfer had also played an important role in the metal ions sorption by the dried biomass of* Kappaphycus* sp. [13].

### 3.7. Thermodynamic Studies

**Figure 4:**
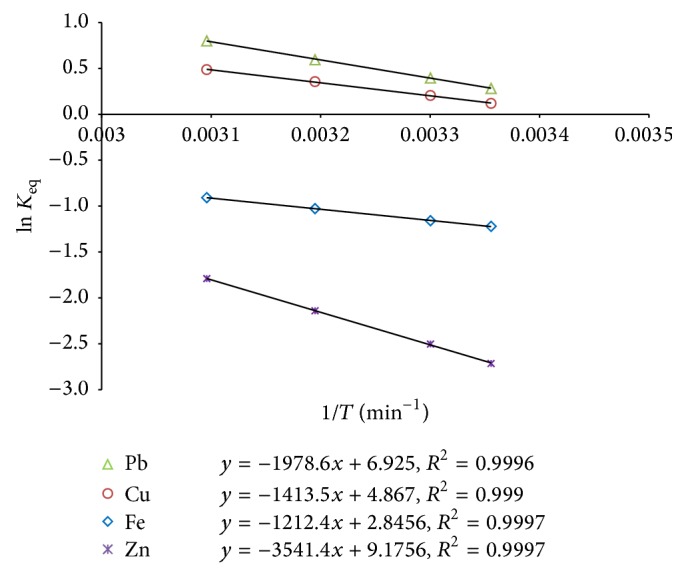
Van't Hoff plots for the metal ions biosorption onto* Kappaphycus* sp. dried biomass.

The values of the thermodynamic parameters are shown in Table 6. The linearized Van't Hoff plots of ln (*K*_eq_) versus 1/*T* are represented in Figure 4. The negative values of Δ*G*° indicate that the thermodynamic process was spontaneous and feasible for all the tested metal ions [35]. Moreover, the increase in negative Δ*G*° values with an increase in temperature shows an increased feasibility of adsorption at higher temperature, which is corroborative to the earlier reports [48, 52, 59].

The positive values of enthalpy change (Δ*H*°) suggest endothermic nature of the metal adsorption process [13, 51, 59, 63, 64]. In addition, the extent of enthalpy value gives indicative information on the type of biosorption, which can be either physical or chemical. The enthalpy change (Δ*H*°) in the range of 2.1–20.9, 20.9–80.0, and 80.0–418.4 kJ mol^−1^ is indicative of physisorption, physisorption together with chemisorptions, and chemisorptions, respectively [48]. Based on the values of Δ*H*°, it can be presumed that the biosorption process took place physically for all the tested metal ions. This was also supported by D-R isotherm results with the *E* (< 8 kJ mol^−1^) values of the metal ions (Table 3). Further, positive values of entropy change (Δ*S*°) are suggestive of increased randomness at the solid-solution interface during the biosorption process of the metal ions on the active sites of the biosorbent [59].

### 3.8. Error Function

In the real-world, data samples from each experiment in a series of experiments differ due to measurement error affecting data precision. In order to ensure accurate measurement results, statistical error function is the measure to compensate data errors [65]. Hence, the isotherm and kinetic data were further analyzed using nine error functions in order to test the fitness of the models. Lower value of* SSE*,* ARE*,* HYBRID*,* EABS*,* MPSD*, *χ* ^2^, Δ*q*(%), and* RMSE* and higher value of *r* ^2^ indicate the best fit of the model.

The correlation of regression (*R* ^2^) for the adsorption isotherm models (Table 3) suggests that Pb^2+^, Fe^2+^, and Zn^2+^ follow the Langmuir model while Cu^2+^ follows the Temkin model accurately. The error functions of the isotherm data (Table 7) suggest that the Temkin model provides the best fit to the experimental data. Again, the correlation of regression (*R* ^2^) for the kinetic models (Table 5) shows that PSO is the best fit model. But the error functions of the kinetic data (Table 8) suggest that the best fit of the kinetic models is intraparticle diffusion. It is, therefore, strongly suggested that the regression coefficient (*R* ^2^) is not an appropriate method for comparing the best fitting of the isotherm and kinetic models; rather some forms of error analysis could be a better criterion for avoiding data errors.

### 3.9. FTIR Spectral Analysis

**Figure 5:**
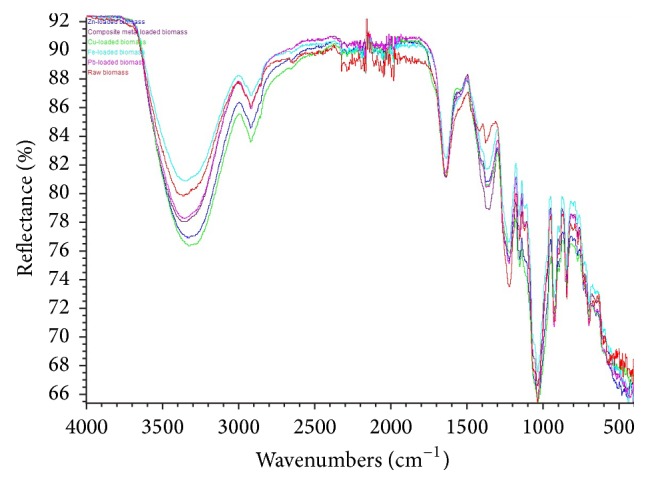
FTIR spectra of the functional groups in* Kappaphycus* sp. biomass before and after biosorption of the metal ions.

The FTIR spectra of* Kappaphycus* sp. (Figure 5) consist of a number of absorption peaks which indicate complex nature of the biomass. The strong broad peak observed at 3358.1 cm^−1^ in the raw biomass corresponds to O–H group from cellulose and N–H groups from proteins in the seaweed [66]. In the spectra other dominant peaks were observed at wavenumbers (cm^−1^) 2917.1, 1636.5, 1375.0, 1220.1, 1155.1, 1035.2, 924.0, and 842.9 which are characterized to the asymmetric C–H stretching vibrations of the aliphatic groups [67], C=O stretching vibration of carboxylate groups [48, 60], asymmetric stretching of –SO_3_^−^ bonds in sulfonic acid [48], C=O stretching vibration of carboxylate groups [48, 60], symmetric stretching of –SO_3_^−^ bonds in sulfonic acid [48], C–O stretching vibration of carboxyl groups [60], S–O stretching [60], and S=O stretching bands of sulfonate groups [48], respectively.

After biosorption of Pb^2+^ the peaks were shifted to 3355.9, 2917.8, 1638.8, 1370.4, 1222.0, 1154.1, 1033.5, 924.8, and 844.5 cm^−1^, respectively. After biosorption of Cu^2+^ the peaks were changed to 3324.7, 2919.9, 1638.3, 1370.5, 1216.0, 1153.3, 1032.7, 925.3, and 845.9 cm^−1^, respectively. The peaks after Fe^2+^ biosorption were changed to 3351.9, 2916.8, 1637.2, 1369.2, 1223.9, 1154.5, 1032.1, 925.2, and 844.2 cm^−1^, respectively. After biosorption of Zn^2+^ the peaks were shifted to 3328.4, 2918.1, 1636.9, 1370.3, 1221.1, 1154.2, 1030.9, 924.7, and 845.3 cm^−1^, respectively. In the quaternary system, the peaks after biosorption were shifted to 3348.3, 2918.0, 1636.1, 1355.1, 1224.0, 1154.9, 1033.1, 924.9, and 845.1 cm^−1^, respectively. The significant change in the intensity of the peaks shows interaction between the metal ions and the functional groups. Because intensity depends on change in dipole moment and total number of functional groups present on biosorbent surface. Therefore, it can be concluded that the carboxylic, sulfonic acid, and sulfonate groups of* Kappaphycus* sp. dried biomass are involved in the biosorption of the metal ions.

## 4. Conclusion

In the present study, we examined adsorption of four heavy metal ions such as Pb^2+^, Cu^2+^, Fe^2+^, and Zn^2+^ onto the dried biomass of the red seaweed* Kappaphycus* sp. from Malaysia. The adsorption isotherm data for the metal ions fitted well with the Temkin model followed. Kinetic data for all the metal ions can be best described by the intraparticle diffusion model. Adsorption process was feasible, spontaneous, and endothermic in nature. We strongly suggest that analysis of error functions is a better criterion for validating isotherm and kinetic models in order to evaluate adsorptive behaviour of a typical adsorbent using linear method.

Heavy metal adsorption process onto the dried biomass of* Kappaphycus* sp. was the complex one involving more than one mechanism. Both homogeneous and heterogeneous active sites were found to exist in the dried biomass. The FTIR study revealed the presence of carboxylic, sulfonic acid, and sulfonate groups in the cell wall matrix of the biomass that was involved in the adsorption of the metal ions. The dried biomass of* Kappaphycus* sp. may be used as a low-cost biosorbent for removal of heavy metal ions from aqueous solutions. Further study is warranted to evaluate the potentiality of the biosorbent for heavy metal removal from the real environment.

## Figures and Tables

**Table 1:** Equations of the isotherm, kinetic, and thermodynamic models used in the study.

Model	Equation	Reference
Langmuir	$\frac{C_{e}}{q_{e}}=\frac{1}{K_{L}q_{m}}+\frac{C_{e}}{q_{m}}$	[25]
	$R_{L}=\frac{1}{\left(1+K_{L}C_{0}\right)}$	[26]

Freundlich	$\log q_{e}=\log K_{F}+\frac{1}{n}\log C_{e}$	[27]

Temkin	$q_{e}=\left(\frac{R_{T}}{b_{T}}\right)\ln A_{T}+\left(\frac{R_{T}}{b_{T}}\right)C_{e}$	[28]

D-R	ln⁡(*q*_*e*_) = ln⁡(*q*_*m*_) − *B*_*D*_ε^2^	[29]
	$\varepsilon =RT\ln\left(1+\frac{1}{C_{e}}\right)$	
	$E=\frac{1}{\sqrt{\left(-2B_{D}\right)}}$	[30]

PFO	$\log\left(q_{e}-q_{t}\right)=\log q_{e}-\frac{k_{1}t}{2.303}$	[31]

PSO	$\frac{t}{q_{t}}=\frac{1}{k_{2}{q_{e}}^{2}}+\frac{t}{q_{e}}$	[32]
	*h* = *k*_2_ *q*_*e*_^2^	
	$t_{1/2}=\frac{1}{k_{2}q_{e}}$	

Elovich	$\frac{dq_{t}}{d_{t}}=\alpha \exp\left(-{\beta q}_{t}\right)$	[28]
	$q_{t}=\frac{1}{\beta }\ln\left(\alpha \beta \right)+\frac{1}{\beta }\ln\left(t\right)$	[33]

IpD	*q*_*t*_ = *k*_*d*_ *t* ^0.5^ + *C*	[34]

Thermodynamics	Δ*G*° = −*RT*ln⁡*K*_eq_	[35]
	Δ*G*° = Δ*H*° − *T*Δ*S*°	
	$\ln K_{\text{eq}}=\frac{\Delta S^{{}^{\circ}}}{R}-\frac{\Delta H^{{}^{\circ}}}{RT}$	
	$K_{\text{eq}}=\frac{q_{e}}{C_{e}}$	

*C*_0_ (mg L^−1^): adsorbate initial concentration, *C*_*e*_ (mg L^−1^): adsorbate equilibrium concentration, *q*_*e*_ (mg g^−1^): observed biosorption capacity at equilibrium, *q*_*m*_ (mg g^−1^): maximum biosorption capacity, *K*_*L*_ (L mg^−1^): Langmuir constant related to the energy of adsorption, (*R*_*L*_): a dimensionless constant, known as separation factor, *K*_*F*_ (mg g^−1^) (L mg^−1^)^1/*n*^: Freundlich isotherm constant related to the sorption capacity, *n*: a constant which gives an idea of the grade of heterogeneity, *R* (8.314 J mo^−1^): universal gas constant, *T* (°K): absolute temperature, *A*_*T*_ (L mg^−1^): equilibrium binding constant corresponding to the maximum binding energy, *b*_*T*_ (J mol^−1^): Temkin constant related to heat of sorption, *B*_*D*_ (mol^2^ kJ^−2^): Dubinin-Radushkevich isotherm constant, *ε*: Polanyi potential related to the equilibrium concentration, *E* (kJ mol^−1^): mean free energy of biosorption, *q*_*t*_ (mg g^−1^): equilibrium adsorption uptake at time, *t*, *k*_1_ (min^−1^): pseudo-first-order rate constant of adsorption, *k*_2_ (g mg^−1^ min^−1^): pseudo-second-order rate constant of adsorption, *h* (mg g^−1^ min^−1^): initial adsorption rate, *t*_1/2_ (min): half-adsorption time, *α* (mg g^−1^ min^−1^): initial adsorption rate constant, *β* (g mg^−1^): desorption constant, *C* (mg g^−1^): boundary layer diffusion effect, *k*_*d*_ (mg g^−1^ min^−0.5^): rate constant for intraparticle diffusion, Δ*G*° (kJ mol^−1^): change in Gibbs free energy, Δ*H*° (kJ mol^−1^): change in enthalpy, Δ*S*° (kJ mol^−1^ K^−1^): change in entropy, and *K*_eq_: thermodynamic equilibrium constant.

**Table 2:** Equations of the error function used in the study.

Equation	Reference
SSE = ∑_*i*=1_^*n*^(*q*_*e*,calc_ − *q*_*e*,exp⁡_)^2^	[36]

$\text{ARE}=\frac{100}{N}\sum_{i=1}^{n}\left|q_{e,\exp}-q_{e,\text{calc}}\right|$	[36]

$\text{HYBRID}=\frac{100}{\left(N-P\right)}\sum_{i=1}^{n}\frac{\left(q_{e,\exp}-q_{e,\text{calc}}\right)}{q_{e,\exp}}$	[36]

EABS = ∑_*i*=1_^*n*^|*q*_*e*,exp⁡_ − *q*_*e*,calc_|	[36]

$\text{MPSD}=100\sqrt{\left(\frac{1}{N-P}\right)\sum_{i=1}^{n}{\left(\frac{q_{e,\exp}-q_{e,\text{calc}}}{q_{e,\exp}}\right)}^{2}}$	[36]

$\Delta q\;\left(\%\right)=100\sqrt{\frac{1}{N-1}\sum_{i=1}^{n}{\left(\frac{q_{e,\exp}-q_{e,\text{calc}}}{q_{e,\exp}}\right)}^{2}}$	[37]

$r^{2}=\frac{\sum_{i=1}^{n}{\left(q_{e,\text{calc}}-\overset{-}{q_{e,\exp}}\right)}^{2}}{\sum_{i=1}^{n}{\left(q_{e,\text{calc}}-\overset{-}{q_{e,\exp}}\right)}^{2}+\sum_{i=1}^{n}{\left(q_{e,\text{calc}}-q_{e,\exp}\right)}^{2}}$	[38]

$\chi^{2}=\sum_{i=1}^{n}\frac{{\left(q_{e,\exp}-q_{e,\text{calc}}\right)}^{2}}{q_{e,\text{calc}}}$	[39]

$\text{RMSE}=\sqrt{\frac{1}{N-2}\sum_{i=1}^{n}{\left(q_{e,\exp}-q_{e,\text{calc}}\right)}^{2}}$	[40]

*q*_*e*,exp⁡_ (mg g^−1^): value obtained from the batch experiment, *q*_*e*,calc_ (mg g^−1^): calculated value from the isotherm for corresponding *q*_*e*,exp⁡_, $\overset{-}{q_{e,\exp}}$ (mg g^−1^): mean of *q*_*e*,exp⁡_, *N*: number of observations in the experimental isotherm, and *P*: number of parameters in the respective model.

**Table 3:** Biosorption isotherm model parameters for the metal ions biosorption onto *Kappaphycus* sp. dried biomass.

Model	Parameter	Metal ion			
		Pb^2+^	Cu^2+^	Fe^2+^	Zn^2+^
Langmuir	*q*_*m*_ (mg g^−1^)	22.17	19.49	16.92	16.23
	*K*_*L*_ (L mg^−1^)	0.0676	0.0728	0.073	0.0561
	*R*_*L*_	0.37–0.07	0.35–0.06	0.35–0.06	0.41–0.08
	*R* ^2^	0.995	0.993	0.997	0.986

Freundlich	*K*_*F*_ (mg g^−1^) (L mg^−1^)^1/*n*^	3.836	4.000	3.491	3.164
	*n*	2.744	3.091	3.104	3.121
	*R* ^2^	0.986	0.984	0.934	0.938

Temkin	*A*_*T*_ (L mg^−1^)	1.037	1.397	1.228	1.056
	*b*_*T*_ (kJ mol^−1^)	596.703	720.350	800.999	853.158
	*R* ^2^	0.991	0.995	0.989	0.967

D-R	*q*_*m*_ (mg g^−1^)	15.58	14.33	13.22	12.16
	*B*_*D*_ (mol^2^ kJ^−2^)	2.284	2.224	4.773	5.768
	*R* ^2^	0.773	0.799	0.894	0.865
	*E* (kJ mol^−1^)	0.468	0.474	0.324	0.294

**Table 4:** Maximum adsorption capacity of heavy metals by some low-cost sorbents.

Low-cost sorbent	Adsorption capacity (mg g^−1^)				Reference
	Pb^2+^	Cu^2+^	Fe^2+^	Zn^2+^	
Activated carbon from coconut	4.56	—	—	—	[41]
Activated carbon from seed hull of the palm tree	3.58	—	—	—	[41]
Epichlorohydrin-crosslinked chitosan	34.13	35.46	—	10.21	[42]
Hazelnut husk	13.05	6.645	—	—	[43]
Natural muscovite	0.63	0.618	—	—	[44]
Kaolinite	7.75	4.42	—	4.95	[45]
Modified orange peel	73.53	15.27	—	—	[46]
Coconut tree sawdust	25.00	3.89	—	23.81	[47]
Sugarcane bagasse	21.28	3.65	—	40.00	[47]
*Kappaphycus* sp.	22.27	19.46	17.09	16.78	Present study

**Table 5:** Kinetic model parameters for the metal ions biosorption onto *Kappaphycus* sp. dried biomass.

Model	Parameter	Metal ion			
		Pb^2+^	Cu^2+^	Fe^2+^	Zn^2+^
	*q*_*e*,exp⁡_ (mg g^−1^)	2.106	2.0467	1.3554	0.5255

Pseudo-first-order	*k*_1_ (min^−1^)	0.035	0.0435	0.0454	0.0329
	*q*_*e*,calc_ (mg g^−1^)	0.1456	0.1162	0.53	0.1262
	*R* ^2^	0.986	0.997	0.995	0.974

Pseudo-second-order	*k*_2_ (g mg^−1^ min^−1^)	0.6133	0.9047	0.1771	0.5968
	*q*_*e*,calc_ (mg g^−1^)	2.1159	2.0559	1.3999	0.535
	*h* (mg g^−1^ min^−1^)	2.7457	3.8241	0.3471	0.1708
	*t*_1/2_ (min)	0.7706	0.5376	4.0335	3.1322
	*R* ^2^	1.000	1.000	0.9999	0.9995

Elovich	*α* (mg g^−1^ min^−1^)	5.67E + 21	1.62E + 22	62.4735	3276.307
	*β* (g mg^−1^)	27.6243	28.8184	7.9618	27.0124
	*R* ^2^	0.9845	0.9318	0.9753	0.8817

Intraparticle diffusion	*k*_*d*_ (mg g^−1^ min^−0.5^)	0.0054	0.0099	0.0311	0.0112
	*C* (mg g^−1^)	2.0457	1.9636	1.0597	0.4142
	*R* ^2^	0.985	0.988	0.965	0.997

**Table 6:** Thermodynamic parameters for the metal ions biosorption onto *Kappaphycus* sp. dried biomass.

Metal ion	Δ*H*°	Δ*S*°	Δ*G*° (kJ mol^−1^)				*R* ^2^
	(kJ mol^−1^)	(kJ mol^−1^ K^−1^)	298° K	303° K	313° K	323° K	
Pb^2+^	16.4501	57.5745	−0.7071	−0.9950	−34.4709	−35.0466	0.999
Cu^2+^	11.7518	40.4642	−23.8102	−24.0125	−24.4171	−24.8218	0.999
Fe^2+^	10.0799	23.6583	−17.1301	−17.2484	−17.4849	−17.7215	0.999
Zn^2+^	29.4432	80.7755	−53.5143	−53.9182	−54.7259	−55.5337	0.999

**Table 7:** Error function data of the isotherm models.

Metal ion	Isotherm model	Error function								
		SSE	ARE	HYBRID	EABS	MPSD	*r* ^2^	*χ* ^2^	Δ*q* (%)	RMSE
Pb^2+^	Langmuir	4.4347	6.7140	9.3996	4.2033	34.7188	0.9774	0.7927	12.6314	0.94177
	Freundlich	4.9053	4.3967	6.1554	4.3317	23.7746	0.9739	0.2681	5.5740	0.99049
	Temkin	1.4457	3.9218	5.4905	2.7612	17.4775	0.9911	0.1572	5.6824	0.53772
	Dubinin-Radushkevich	58.3244	17.0502	23.8703	16.5533	92.0214	0.5995	3.8955	23.8712	3.41539

Cu^2+^	Langmuir	5.7968	8.5817	12.0143	5.3808	39.3515	0.9629	1.0282	14.1340	1.07674
	Freundlich	2.2807	4.0093	5.6130	3.2226	18.7862	0.9826	0.1747	5.2390	0.67538
	Temkin	0.5693	2.1797	3.0515	1.7002	10.1114	0.9951	0.05078	3.0016	0.33742
	Dubinin-Radushkevich	37.5579	14.6298	20.4817	13.3928	75.4605	0.6420	2.7153	19.7375	2.74073

Fe^2+^	Langmuir	2.3495	5.4061	7.5685	3.6433	22.5599	0.9760	0.2778	7.1832	0.68549
	Freundlich	5.1830	7.6257	10.6759	5.2617	32.1906	0.9476	0.5282	10.0870	1.01814
	Temkin	0.9198	3.0795	4.31124	1.6626	15.7644	0.9885	0.1240	5.5745	0.42890
	Dubinin-Radushkevich	14.9728	10.1415	14.1981	8.5372	48.9560	0.7916	1.1939	12.9975	1.73048

Zn^2+^	Langmuir	6.4809	9.6783	13.5496	6.0631	38.6885	0.9325	0.8625	12.6706	1.13850
	Freundlich	3.0423	5.2872	7.4021	3.0394	27.2346	0.9602	0.4149	9.0515	0.78004
	Temkin	2.2050	4.9041	6.8658	3.2227	21.6034	0.9674	0.2471	6.6271	0.66408
	Dubinin-Radushkevich	16.4718	10.9989	15.3985	8.8422	51.8019	0.7325	1.3963	13.8314	1.81504

**Table 8:** Error function data of the kinetic models.

Metal ion	Kinetic model	Error function								
		SSE	ARE	HYBRID	EABS	MPSD	*r* ^2^	*χ* ^2^	Δ*q* (%)	RMSE
Pb^2+^	Pseudo-first-order	25.7891	92.8427	129.9800	13.4342	157.948	0.49997	178.767	100.288	2.27108
	Pseudo-second-order	0.003366	0.56776	0.79486	0.08086	1.82415	0.80927	0.00171	1.17070	0.02595
	Elovich	9.30E − 05	0.16020	0.22428	0.02312	0.30064	0.98456	4.52E − 05	0.19128	0.00431
	Intraparticle diffusion	2.39E − 06	0.03899	0.11698	0.00245	0.10672	0.98437	1.14E − 06	0.05210	0.00155

Cu^2+^	Pseudo-first-order	25.3581	94.2342	131.928	13.3208	158.455	0.49992	220.398	101.787	2.25202
	Pseudo-second-order	0.00015	0.16029	0.22440	0.02232	0.38712	0.97869	7.51E − 05	0.25151	0.00544
	Elovich	0.00040	0.33223	0.46512	0.04693	0.62860	0.93190	0.00020	0.40416	0.00893
	Intraparticle diffusion	3.29E − 06	0.05087	0.15262	0.00310	0.12720	0.98718	1.62E − 06	0.06311	0.00181

Fe^2+^	Pseudo-first-order	3.49809	56.1628	78.6279	4.90190	74.5604	0.49752	6.56855	60.8816	0.83643
	Pseudo-second-order	0.00400	1.35554	1.89776	0.10685	2.72686	0.95949	0.00390	2.40267	0.02828
	Elovich	0.00181	1.13760	1.59264	0.09939	1.68403	0.97531	0.00141	1.36581	0.01902
	Intraparticle diffusion	0.00031	0.66725	1.33449	0.03436	1.08937	0.96472	0.00024	0.78427	0.01236

Zn^2+^	Pseudo-first-order	0.92442	73.8117	103.336	2.53393	61.3141	0.49932	7.65727	79.9178	0.42998
	Pseudo-second-order	0.00279	2.59220	3.62908	0.08525	3.48380	0.77805	0.00673	4.68899	0.02363
	Elovich	0.00064	1.63635	2.29089	0.05555	1.62276	0.88172	0.00133	2.12691	0.01133
	Intraparticle diffusion	3.87E − 06	0.18119	0.36239	0.00362	0.19597	0.99645	7.68E − 06	0.22567	0.00139
